# Sex hormone-binding globulin is a valuable diagnostic indicator of gestational diabetes mellitus

**DOI:** 10.4314/gmj.v58i1.8

**Published:** 2024-03

**Authors:** Basil Bruno, Myke-Mbata K Blessing, Mba N Izuchukwu, Gbaa T Terry, Dogoh Faeren

**Affiliations:** 1 Department of Chemical Pathology, Benue State University, Makurdi, Nigeria; 2 Department of Chemical Pathology, Nile University, Abuja, Nigeria; 3 Department of Life Sciences, Manchester Metropolitan University, Manchester, United Kingdom

**Keywords:** Diagnostic indicator, Gestational diabetes, Oral Glucose Tolerance Test (OGTT), Sex Hormone-Binding Globulin (SHBG)

## Abstract

**Objective:**

To assess the performance of the Sex Hormone-Binding Globulin (SHBG) assay as a diagnostic indicator of Gestational Diabetes Mellitus (GDM) in the study population.

**Design:**

Analytical cross-sectional study

**Setting:**

Hospital-based, Benue State University Teaching Hospital (BSUTH), Makurdi, Nigeria.

**Participants:**

Women with singleton pregnancies at 24 to 28 weeks gestational age attending Antenatal care at BSUTH, Makurdi.

**Intervention:**

Serum SHBG levels were assayed by ELISA during a diagnostic 75-gram Oral Glucose Tolerance Test (OGTT) for assessment of GDM in the cohort of consecutively selected participants who met the inclusion criteria.

**Main Outcome Measures:**

Serum levels of SHBG and presence of GDM in the participants.

**Result:**

Serum SHBG was significantly negatively correlated (rpb = − 0.534, p-value < 0.001) with the presence of GDM. It had an area under the ROC curve of 0.897 (95% Confidence Interval = 0.858–0.935; p-value < 0.001). A cut-off value of 452.0 nmol/L indicative of GDM had a diagnostic odds ratio of 21.4 in the study population.

**Conclusion:**

SHBG is a valuable diagnostic indicator for GDM in the study population.

**Funding:**

None declared

## Introduction

Sex hormone binding globulin (SHBG) is a large homodimeric glycoprotein synthesized mostly in the liver. It binds to estrogens and androgens, serving as their transport protein aside from albumin, thereby influencing the bioavailability of the hormones.^1^ In pregnancy, SHBG synthesis can increase 5- to 10-fold as a result of activation by high estrogen levels. Thus, a normal pregnancy level of SHBG is usually elevated, and this protects the mother from exposure to fetal androgens that escape metabolism by the placenta.^2^

Altered SHBG levels have been associated with polycystic ovarian syndrome, Cushing's syndrome, hypothyroidism, acromegaly, obesity, use of anabolic steroids, hyperthyroidism, oral contraceptives, anorexia nervosa, cirrhosis and Type 2 Diabetes Mellitus (T2DM).^3^–^6^ Crucial to this study is its relationship with T2DM, where a reduction in its serum levels increases the probability of the disease.^3^

This SHBG reduction, alongside certain genetic polymorphisms, leads to and is strongly associated with insulin resistance and, consequently, T2DM.^3^,^7^ Pregnancy-induced impairment of glucose tolerance as a result of pancreatic beta cell dysfunction on a background of increasing insulin resistance has been identified as the pathologic hallmark of gestational diabetes mellitus (GDM),^8^ hence the association between GDM and SHBG.

Gestational diabetes mellitus is defined as any degree of glucose intolerance with onset or first recognition during pregnancy.^9^,^10^ It is diagnosed using the oral glucose tolerance test (OGTT), usually in the 24 to 28 weeks of pregnancy or with a fasting plasma glucose ≥ 5.1 mmol/L anytime in the course of pregnancy.^11^ The OGTT is currently the gold standard reference test for GDM.

However, its diagnostic reliability is limited by concerns of reproducibility and accuracy due to poor patient tolerability, time and cost, fasting and changes in dietary habits prior to testing,^12^–^16^ as well as lack of consensus on timing, procedure, and optimal diagnostic cut-points.^17^ These limitations, especially in situations where patients quit as a result of poor tolerability, may occasionally leave clinicians with no other option but to resort to relying on their often limited clinical judgments.

Many biomarkers have been evaluated as potential alternative diagnostic markers of GDM against the OGTT with variable outcomes.^13^ Some of these markers are implicated in the pathogenesis of the disease. One such is SHBG which has been a subject of extensive studies due to its strong association with GDM and its ability to predict GDM development^18^–^21^ even in similar cohorts from the present study population.^22^,^23^ Considering the established pathological links between SHBG and GDM,^8^ as well as its ability to predict the disease, the investigators hypothesize that SHBG may be a valuable tool for diagnosing GDM. Few studies have evaluated its role in the diagnosis of the disease, but they mostly involved non-African populations.^24^,^25^ This study is aimed at assessing the performance of SHBG against the OGTT as a diagnostic indicator of GDM in the study population.

## Methods

### Ethical considerations

Approval for this study was obtained from the Health Research Ethics Committees of Benue State University Teaching Hospital (BSUTH), Makurdi [registration code: BSUTH/MKD/HREC/2013B/2018/0024] as a component of a broader study. Informed written consent was obtained from each patient before recruitment into the study. Number codes were allotted to each recruited participant to ensure confidentiality throughout the study. Clinical data and test results from the participants were also kept confidential by locking them in secured spaces.

### Study design and setting

This was a hospital-based, analytical cross-sectional study conducted at the antenatal clinic of BSUTH, in Markurdi, North-central Nigeria between June 2018 and September 2020 (15 months), specifically focusing on outpatient participants. Women who met the inclusion criteria were consecutively recruited as participants in the study. The minimum sample size was calculated based on a previously reported prevalence of 8.3% in the area,^26^ and adjusted for a 10% non-response rate, resulting in a target sample size of 130. Participants who appeared for OGTT and completed the procedure (n=306) were included in the statistical analysis and those with normal pregnancy (non-GDM; n=252) formed the control group.

Participants included in this study were women at 24 to 28 weeks of gestation with singleton pregnancies. The gestational age of the participants was preferably calculated based on their last menstrual period and first-trimester ultra-sound scan results in line with the current modalities for determining gestational age in resource-poor regions like the study area. Women with known diabetes mellitus, and hypertension, were acutely or chronically ill or had any conditions which alter plasma SHBG concentrations (e.g., liver disease, malnutrition, HIV infection, thyroid disease, or on medications like steroids, progestins, anticonvulsants, etc.) were excluded. These details were obtained from the patients' folders as well as directly from the participants during the administration of the research proforma.

### Data collection

Data on relevant maternal clinical and demographic characteristics, as well as anthropometric measurements of consenting participants, were obtained by use of a validated and structured study proforma.

### Testing for GDM (24 – 28 weeks OGTT)

Participants were subjected to OGTT with an oral load of 75 grams of anhydrous glucose. Patient preparation involved ensuring they maintained their regular daily dietary intake of approximately 150 – 200 grams of carbohydrate and routine physical activities for at least 3 days before testing, and an overnight fast of approximately 8-to 12 hours on the morning of OGTT. Samples for plasma glucose assay were collected into tubes with fluoride oxalate, separated, and analyzed within 20 minutes of collection in batches.

Diagnosis of GDM was according to the updated international diagnostic criteria based on the International Association of Diabetes in Pregnancy Study Group (IADPSG) diagnostic guideline for universal testing^27^ between 24 – 28 weeks gestation. Diagnosis of GDM was made when fasting plasma glucose level ≥ 5.1mmol/L, and/or 1-hour post-75g oral glucose load blood glucose level ≥ 10.0mmol/L, and/or 2-hour post-75g oral glucose load blood glucose level ≥ 8.5mmol/L.

### SHBG Assay

A concurrent fasting venous blood sample was collected during OGTT from each participant into plain vacutainer tubes for SHBG assay. These samples were centrifuged at 3000 rpm for 10 min after clotting and retraction, separated, and stored frozen at a −85°C freezer until assayed. Measurement of serum SHBG was by quantitative sandwich enzyme-linked immunosorbent assay (ELISA) technique. The assay kit was the SHBG AccuBind ELISA test system^28^ supplied by Monobind Inc., California, USA. Its analytical sensitivity was 0.0122nmol/L.

### Statistical analysis

Data from the study participants were analyzed using the Statistical Package for Social Sciences (SPSS) version 25 from IBM Corporation, Armonk, New York, United States. Categorical variables like family history of diabetes mellitus, foetal macrosomia, and GDM development were dichotomized, while quantitative variables, such as the SHBG level, Body Mass Index (BMI), and age were represented by their mean and analyzed as continuous variables. The mean serum SHBG levels were compared between participants with and without GDM by using Student's t-test while a similar comparison for discrete independent variables was done using the chi-square test. The relationship between SHBG and GDM was determined using the point-biserial correlation^29^ (significant at the 0.05 level).

The performance of serum SHBG level as an indicator of GDM and the determination of its optimal cut-off point for detecting the disease was determined by receiver operator characteristic (ROC) curve analysis, and the area under the curve (AUC) served as a measure of the diagnostic performance and clinical utility of the test.

## Results

**Figure 1:**
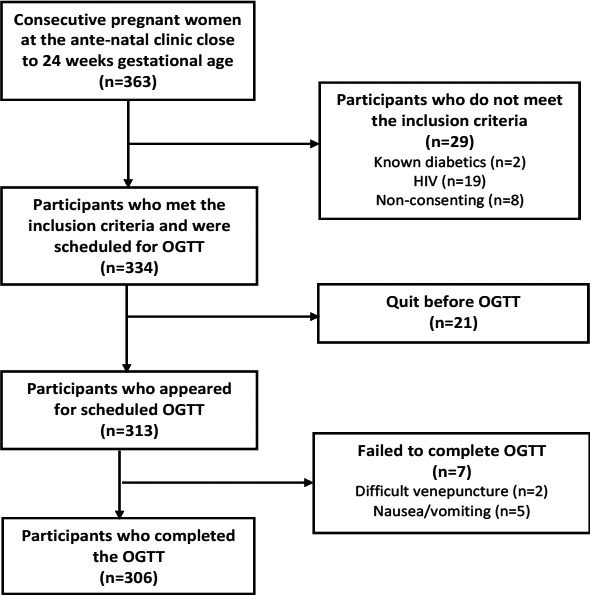
Flow chart showing recruitment of participants

A total of 334 pregnant women expected to be within 24 – 28 weeks of gestational age at a scheduled OGTT time were recruited into the study but only 306 completed the test procedure and were included in the statistical analysis (Figure 1). Amongst participants who completed the procedure, 54 (17.6%) were diagnosed with GDM in the 75g OGTT while 252 (82.4%) were without the disease.

The clinical and demographic characteristics of the study participants are shown in Table 1. Statistically significant differences were seen in the GDM versus Non-GDM participants with regards to mean age (31.3 ± 4.7 years versus 28.7 ± 4.7 years; p = 0.001), parity (n = 12 versus n = 92; p = 0.44), history of first-degree relations with DM (n = 12 versus n = 42; p = 0.001), previous history of multiple pregnancies (n = 8 versus n = 13; p = 0.011) or foetal macrosomia (n = 19 versus n = 43; p = 0.033). However, no significant differences in ethnicity, educational status, religion, blood pressure, BMI, previous history of perinatal loss, or pre-eclampsia were noted.

**Table 1:** Maternal demographic and clinical characteristics at 24 – 28 weeks gestational age among GDM and non-GDM participants (percentages in brackets)

Maternal Characteristics	Total (n=306) Mean±SD or N (%)	GDM (n=54) Mean±SD or N (%)	Non-GDM (n=252) Mean±SD or N (%)	p-value
**Age groups (years)**	**29.1 ± 4.8**	**31.3 ± 4.7**	**28.7 ± 4.7**	**.000** *
**Ethnic groups**				**.093**
***Tiv***	**168 (54.9)**	**37 (68.5)**	**131 (52.0)**	
***Idoma***	**67 (21.9)**	**6 (11.1)**	**61 (24.2)**	
***Igbo***	**38 (12.4)**	**7 (13.0)**	**31 (12.3)**	
***Others***	**33 (10.8)**	**4 (7.4)**	**29 (11.5)**	
**Educational status**				**.157**
***Uneducated***	**74 (24.2)**	**15 (27.8)**	**59 (23.4)**	
***Primary***	**62 (20.3)**	**16 (29.2)**	**46 (18.3)**	
***Secondary***	**70 (22.9)**	**9 (16.7)**	**61 (24.2)**	
***Tertiary***	**100 (32.7)**	**14 (25.9)**	**86 (34.1)**	
**Religion**				**.590**
***Christian***	**259 (84.6)**	**47 (87.0)**	**212 (84.1)**	
***Muslim***	**47 (15.4)**	**7 (12.9)**	**40 (15.9)**	
**Parity**				**.044** *
***Primigravida***	**104 (34.0)**	**12 (22.2)**	**92 (36.5)**	
***Multigravida***	**202 (66.0)**	**42 (77.8)**	**160 (63.5)**	
**Blood Pressure**				
***Systolic (mmHg)***	**108.0 ± 10.9**	**108.0 ± 10.9**	**108.0 ± 10.9**	**.994**
***Diastolic (mmHg)***	**72.4 ± 10.9**	**72.8 ± 10.5**	**72.4 ± 11.0**	**.809**
**BMI (kg/m^2^)**	**31.0 ± 1.7**	**31.1 ± 1.7**	**30.8 ± 1.7**	**.220**
**History of First-Degree Relations with DM**	**64 (20.9)**	**22 (40.7)**	**42 (16.7)**	**.000** *
**History of Perinatal Loss**	**39 (12.7)**	**11 (20.4)**	**28 (11.1)**	**.073**
**History of Multiple Pregnancy**	**21 (5.8)**	**8 (14.8)**	**13 (5.2)**	**.011** *
**History of Foetal Macrosomia (birth weight ≥ 4kg)**	**62 (20.3)**	**19 (35.2)**	**43 (17.1)**	**.003** *
**History of Pre-eclampsia**	**16 (5.2)**	**5 (9.3)**	**11 (4.4)**	**.143**

* p-value significant at < 0.05; N = number of participants in subgroup; n = number of participants in main group; SD = Standard Deviation

The mean serum SHBG level at 24 – 28 weeks gesta-tional age of the participants was 534.6 nmol/L (SD ± 141.7). Participants with GDM (n=54) had significantly lower mean SHBG value (359.2 nmol/L (SD ± 113.4)) compared to non-GDM (n=252) participants with a mean value of 572.1 nmol/L (SD ± 116.8) (p = 0.001).

When adjusted for all possible confounders including maternal age, parity, history of first-degree relations with DM, previous multiple pregnancies, and previous macrosomia, the serum level of SHBG at 24 – 28 weeks gestation in the study participants was significantly negatively correlated with the presence of GDM (r_pb_ = −0.534, p = 0.001) diagnosed via 75g OGTT. This implies a strong correlation between GDM diagnosis and lower levels of serum SHBG at 24 – 28 weeks of gestation independent of other risk factors for GDM.

**Figure 2:**
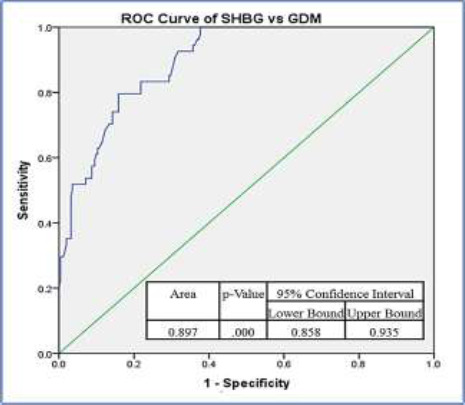
Receiver operator curve for serum SHBG at 24 – 28 weeks and presence of GDM

The performance of serum SHBG as a diagnostic marker of GDM was determined via ROC curve analysis (Area under the curve = 0.897; 95% Confidence Interval = 0.858–0.935; p = 0.001) (Figure 2). Further analysis of the ROC curve was used to determine an optimal cut-off level of 452.0 nmol/L for the serum SHBG diagnostic of GDM development in the study population. At this cut-off point, serum SHBG exhibited a diagnostic sensitivity of 80.1% and a diagnostic specificity of 84.2%. It also had a positive predictive value of 52.0%, a negative predictive value of 95.2%, and a calculated diagnostic odds ratio of 21.4 in the study population.

## Discussion

The disease burden of GDM in the sub-Saharan African region is high, and over the years, there seems to be a progressive rise in the prevalence of the disease.^30^,^31^ A recent systematic review and meta-analysis revealed a prevalence of 16.0% (95% CI: 8.0 – 25.0) in the region^32^ which is consistent with our finding of 17.6% in the index study. This high prevalence can be explained by changes in diagnostic criteria and/or definition of GDM, increasing maternal age and BMI, and other lifestyle changes.^33^ However, the prevalence from the present study may not reflect the true GDM prevalence in the study area as such a finding was not intended in the study design and sampling method.

In this study, advancing age among women in their child-bearing years was an important GDM risk factor that was significantly associated with the disease and the same finding has been reported by previous studies.^26^,^34^ This may be attributed to an age-related increase in inducible nitric oxide synthase (iNOS) expression and *S*-nitrosation of the insulin receptor complex in skeletal muscle which leads to insulin resistance.^35^ Also, previous predictive studies where BMI was assessed in early pregnancy, including those carried out in a similar study population, showed that increased BMI was strongly correlated and significantly associated with GDM.^18^–^23^ This may be a result of its association with increased insulin resistance and levels of inflammatory markers in women with higher BMI.^36^–^38^ However, in this study and other previous studies where maternal BMI was assessed within 24 – 28 weeks of gestational age, BMI had no significant relationship with GDM.^24^,^25^ This may suggest that the association between BMI and GDM weakens as pregnancy progresses possibly as a result of an altered pattern of gestational weight gain in the latter stages.

An important finding from this study is the presence of a negative relationship between serum SHBG and GDM which is consistent with results of previous studies,^22^–^25^,^39^–^41^ and this is due to its strong pathologic association with increased insulin resistance, the hallmark of GDM.^8^,^42^,^43^ Lower levels of SHBG occur in GDM patients because increased lipogenesis accompanying insulin resistance alters hepatocyte nuclear factor 4 alpha (HNF-4α) levels and down-regulates SHBG gene expression.^44^ This established relationship between SHBG and GDM from those studies formed the basis for which serum SHBG was suggested as a valuable tool for the prediction, diagnosis, and monitoring of GDM.

Serum SHBG level was an excellent diagnostic indicator of GDM in the study population with an area under the ROC curve of 0.897 (values between 0.8 – 0.9 are categorized as “good”^45^). In comparison with a cross-sectional case-controlled study involving Saudi Arabian women with GDM diagnosed at 24 – 28 weeks gestation, SHBG assay as a diagnostic test for the disease had a better diagnostic performance than in the present study with a “very good” area under the ROC curve of 0.913.^25^ The findings from these studies involving SHBG have diagnostic performance characteristics comparable to other promising biomarkers like leptin, chemerin/FABP ratio, ficolin3/adiponectin ratio, and ultrasound gestational diabetes score that have demonstrated high sensitivity and specificity for diagnosing GDM in previous studies with adequate sample sizes.^13^ The optimum cut-point for serum SHBG level at 24 – 28 weeks gestation in this study was 452.0 nmol/L and values below this cut-off were diagnostic of GDM. The optimal diagnostic cut-off point from a previous study was remarkably lower.^25^ This may be explained by variances in assay methods, ethnic differences of the populations, and the chosen sample sizes.

The potential use of SHBG for the diagnosis of GDM as against the OGTT is a promising one. It implies that there will be less need for the extensive preparation and stressful procedure involved in OGTT. Also, as a gold standard test procedure for GDM, there are concerns over the reproducibility and accuracy of the OGTT, as well as the absence of standardized alternatives in situations where it is not applicable or where patients fail to complete the procedure. Known barriers to completion of OGTT like inability to tolerate test protocol, social/mental health issues, difficulty keeping track of multiple antenatal appointments, etc,^12^–^16^ would not impact the SHBG test as no special preparations are required. Serum SHBG assay is a simple laboratory test that has no diurnal variations and can be performed in the non-fasting state.^46^ More so, the use of SHBG as a diagnostic indicator of GDM will help exclude the discrepancies in results involved with the application of different guidelines for screening and diagnosis of the disease.^47^

This study involved a one-time point assessment for GDM at 24 – 28 weeks gestational age, and there was no post-partum follow-up OGTT and SHBG assay of the participants to ascertain the role of the biomarker in the monitoring of GDM as suggested in a similar previous study^24^. As such, we recommend further studies with larger sample sizes involving different African populations wherein OGTT and SHBG assay are carried into puerperium to assess post-delivery GDM. Also, given the marked difference between the serum SHBG diagnostic cut-off points between this study and previous studies as highlighted above, we recommend a standardization of the assay methods, as well as a verification of our findings in other sub-Saharan African populations.

## Conclusion

The current diagnostic procedure for GDM (OGTT) is limited by issues of reproducibility and accuracy, as well as other problems associated with the testing process. In this study, serum SHBG was found to be a valuable diagnostic indicator for GDM in the study population, and it may be useful in overcoming some of the challenges inherent in OGTT, especially in situations where there are barriers to the procedure.
